# Flow cytometry assay for the detection of single-copy DNA in human lymphocytes

**DOI:** 10.1093/nar/gkaa515

**Published:** 2020-06-16

**Authors:** Naoki Uno, Norihito Kaku, Yoshitomo Morinaga, Hiroo Hasegawa, Katsunori Yanagihara

**Affiliations:** Department of Laboratory Medicine, Nagasaki University Graduate School of Biomedical Sciences. Nagasaki 852-8501, Japan; Department of Laboratory Medicine, Nagasaki University Graduate School of Biomedical Sciences. Nagasaki 852-8501, Japan; Department of Laboratory Medicine, Nagasaki University Graduate School of Biomedical Sciences. Nagasaki 852-8501, Japan; Department of Laboratory Medicine, Nagasaki University Graduate School of Biomedical Sciences. Nagasaki 852-8501, Japan; Department of Laboratory Medicine, Nagasaki University Graduate School of Biomedical Sciences. Nagasaki 852-8501, Japan

## Abstract

Specific nucleic acid sequences can be detected in individual cells by *in situ* hybridization. However, when very few copies of a target sequence are present per cell, its signal is undetectable by flow cytometry. Although various approaches have been developed to increase fluorescence signals for *in situ* hybridization, flow cytometric detection of specific genomic DNA sequences has not been established. Here, we present a flow cytometry assay for detection of single-copy genomic sequences in human lymphocytes using *in situ* PCR with universal energy transfer-labelled primers.

## INTRODUCTION

Fluorescence *in situ* hybridization (FISH) has been widely used to detect specific nucleic acid sequences in individual cells. Many signal amplification technologies such as branched DNA, rolling circle amplification, and hybridization chain reaction approaches have been developed to increase the signal from oligonucleotide probes and improve imaging of RNA molecules (1–3). Techniques to visualize individual DNA molecules have also advanced (4–6). However, although FISH-based advanced imaging of nucleic acid molecules in individual cells has been achieved, FISH has not been fully adapted on flow cytometry platforms. Fluorescence signals detectable by microscopy are generally too low to be detected by flow cytometry. Although several methods have enabled detection of nucleic acids by flow cytometry (7–10), they target multiple-copy molecules such as mRNA and microRNA. Flow cytometric detection of genomic DNA molecules is challenging because only two or one copies of chromosomal DNA are present in a diploid cell. Thus, a bright signal with an excellent signal-to-noise ratio is required to detect genomic DNA molecules by flow cytometry. A suitable flow cytometry assay capable of detecting single-copy DNA sequences is yet to be established.

To overcome this limitation, we attempted target amplification rather than signal amplification. *In situ* PCR is a target amplification method that amplifies specific DNA sequences in individual cells using reaction mixtures that typically contain digoxigenin-labelled dUTP. Digoxigenin-labelled dUTP is incorporated in PCR products and detected by an anti-digoxigenin antibody (11). *In situ* PCR was initially developed for detection of human immunodeficiency virus type-1 DNA integrated in the human genome by microscopy and flow cytometry in 1992 and 1993, respectively (12,13). Detection of low copy-number targets was anticipated because DNA sequences of interest can be amplified by PCR. However, non-specific amplification has proved problematic for *in situ* PCR, and represents a major barrier to achieving specific detection of target sequences. One group reported the use of a universal energy transfer-labelled primer (UniPrimer) instead of digoxigenin-labelled dUTP to detect *in situ* PCR products by microscopy (14), but the technique has not been developed further. Herein, we used a UniPrimer to optimize an *in situ* PCR protocol and succeeded in developing a flow cytometry assay to detect single-copy DNA in human lymphocytes.

**Figure 1.**
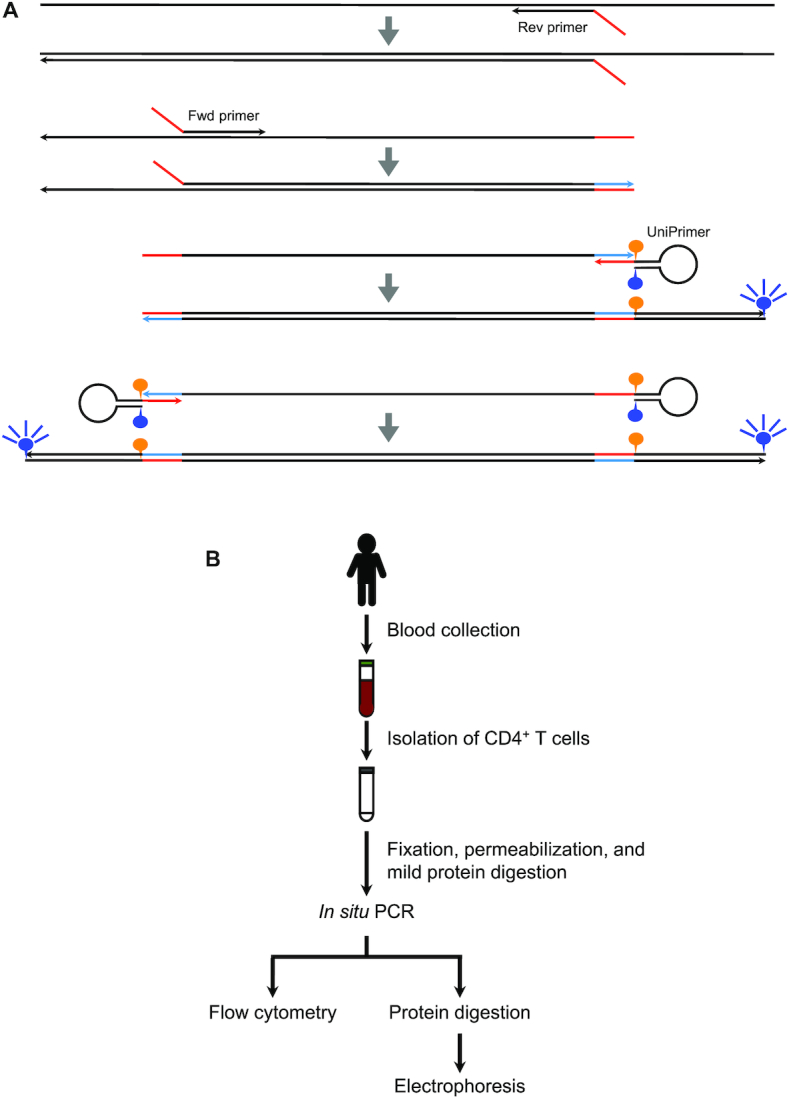
Experimental approach for the detection of specific genomic sequences in human lymphocytes by flow cytometry. (**A**) Schematic diagram of *in situ* PCR using UniPrimers. Arrowheads indicate the 5′→3′ direction of DNA synthesis. Blue and orange circles indicate fluorescein and its quencher, respectively. Both forward and reverse primers contain a tail sequence (red) at their 5′ end. The UniPrimer contains the same sequence at its 3′ end. DNA polymerase extends nucleotides from target-specific primers and synthesizes a sequence complementary to the tail sequence (blue). The UniPrimer anneals to the newly synthesized sequence (blue), gets incorporated into a PCR product, and emits fluorescence because the fluorophore is separated from its quencher in the product. (**B**) Experimental workflow. CD4^+^ T-cells are separated from blood, fixed, and permeabilized. Following mild digestion of cellular proteins, *in situ* PCR is performed to amplify target DNA sequences in individual cells. After PCR, cells are analysed by flow cytometry and gel electrophoresis.

An energy transfer-labelled primer is a fluorescent primer developed for quantitative PCR that does not generate background fluorescence due to its hairpin structure in which the quencher is close to the fluorophore and thus quenches fluorescence emission (15). UniPrimers can be used in addition to target-specific forward and reverse primers when either or both target-specific primers contain a tail sequence that is the same as the 3′ end of the UniPrimer (Figure 1A). UniPrimers do not bind anything during the initial stages of PCR. However, as DNA polymerase extends DNA from target-specific primers and synthesizes new sequences that are complementary to the tail sequence, UniPrimers become incorporated into PCR products, resulting in fluorescence emission because the hairpin structure is unfolded and quenching is no longer possible due to the increased distance between the fluorophore and quencher. UniPrimers subsequently function as typical primers and contribute to amplification, resulting in fluorescence emission. The *in situ* PCR technique is often accompanied by non-specific amplification, even without primers (16). UniPrimers can solve primer-independent amplification because they do not emit fluorescence until target-specific primers anneal to target sequences.

We first attempted to develop a flow cytometry assay to detect human T-cell leukaemia virus type 1 (HTLV-1) viral DNA integrated into the human genome. HTLV-1 is a retrovirus infecting human CD4^+^ T-cells. HTLV-1-infected cells cannot be detected by labelling viral proteins because viral proteins are usually undetectable in infected cells *in vivo* (17). The only appropriate way to detect HTLV-1-infected cells is via identification of viral DNA integrated in the human genome. However, since HTLV-1-infected cells contain only one copy of provirus in most cases (18,19), it is difficult to detect HTLV-1 provirus in single cells. A few studies have reported detection of HTLV-1 provirus by microscopy (20,21). However, flow cytometry-based detection of HTLV-1 provirus has not been achieved. HTLV-1-infected cells are clonally proliferated and cause adult T-cell leukaemia (ATL) in a few cases (17). Pathogenesis of HTLV-1-infected cells *in vivo* is not fully understood, and an effective flow cytometry assay capable of detecting infected cells could address this. We attempted to overcome this limitation and provide a tool for the analysis of thousands of HTLV-1-infected cells.

## MATERIALS AND METHODS

### Sample preparation

Blood was drawn from ATL patients and a healthy individual at Nagasaki University Hospital, and used in HTLV-1 experiments. Blood remaining after routine testing in heparin tubes under approval of the ethical committee of the hospital was used in subsequent experiments (Approval No. 16042538). Blood was collected from a healthy female and male in heparin tubes, and used in *SRY* experiments. CD4^+^ T-cells were separated directly from whole blood using an EasySep Direct Human CD4^+^ T-cell isolation kit (Cat. No. 19662; STEMCELL Technologies) according to the manufacturer's instructions. After counting cells, the appropriate number of cells were placed in tubes and centrifuged at 300 g for 5 min; supernatants were removed; and cells were resuspended in BD Cytofix/Cytoperm fixation and permeabilization solution (Cat. No. 554722; BD Biosciences), and incubated for 30 min at room temperature to fix and permeabilize the cells. We subsequently added 1 ml of 1 × BD Perm/Wash buffer (Cat. No. 554723; BD Biosciences) and left the samples overnight at 4°C. On the following day, cells were centrifuged at 300 g for 5 min; supernatants were removed; and cells were washed once with 1 × BD Perm/Wash buffer, resuspended in 50 μg/ml Proteinase K solution (Cat. No. 03 115 828 001; Roche Diagnostics) diluted in 1 × BD Perm/Wash buffer, and incubated at 55°C or 35°C for 15 min. We subsequently added 1 ml of 1 × BD Perm/Wash buffer, centrifuged cells at 300 g for 5 min, washed cells twice with 1 × BD Perm/Wash buffer, and resuspended cells in reagents for PCR.

### *In situ* PCR

PCR mixtures containing 0.05 U/μl Ex Taq HS (Cat. No. RR006; TaKaRa Bio), 1 × Ex Taq buffer (Cat. No. RR006; TaKaRa Bio), 0.2 mM dNTP mixture (Cat. No. RR006; TaKaRa Bio), 0.5 μM Amplifluor UniPrimer II (Cat. No. S7905; Millipore), and 0.1 μM target-specific forward and reverse primers (Sigma Aldrich Japan) were prepared. Oligonucleotide sequences of target-specific primers are shown in Supplementary Table S1. Cells were resuspended in reagents for PCR in PCR tubes containing 1−2 × 10^5^ cells per 30 μl reaction. PCR was performed at 95°C for 10 min, followed by 50 cycles at 95°C for 10 s, 63°C for 20 s, and 72°C for 40 s, a final extension at 72°C for 5 min, and infinite hold at 4°C.

### Flow cytometry analysis

After PCR, whole PCR assay mixtures were transferred to a FACS tube containing 200−300 μl PBS (Gibco), and data were acquired using a BD FACSCalibur or FACSLyric instrument (BD Biosciences). Approximately 5000 events were acquired and analysed by FCS Express 6 (De Novo Software). FSC/SSC dot plots were displayed, and data acquired in the absence and presence of polymerase were overlaid. We then created gates in overlaid FSC/SSC dot plots and analysed gated events using histograms. Histograms acquired from data obtained in the absence and presence of polymerase were overlaid and normalized based on the number of events. The percentage of positive cells was calculated from the overlaid histograms. A cutoff value of the highest 0.1% of histograms acquired in the absence of polymerase was applied, and all cells in the histogram acquired in the presence of polymerase above this cutoff were considered positive.

### Electrophoresis

After PCR, tubes were centrifuged for 3 min, and 25 μl supernatant was removed, mixed with 10 μl of 200 μg/ml Proteinase K, and incubated at 60°C for 90 min. We subsequently mixed 12 μl samples with 2 μl loading buffer (Nippon Gene), loaded samples onto a 2% agarose gel, performed electrophoresis and detected fluorescein in the gel using an LAS-3000 instrument (Fujifilm). A 4 μl sample of FluoroBand 100 bp Fluorescent DNA ladder (SMOBIO Technology) served as a size marker.

### Evaluation of the limit of blank (LoB) and limit of detection (LoD)

The LoB and LoD were calculated as follows (22).

$$\begin{equation*}{\rm{LoB = mea}}{{\rm{n}}_{{\rm{blank}}}}{\rm{ + 1}}{\rm{.645 \times S}}{{\rm{D}}_{{\rm{blank}}}}\end{equation*}$$

$$\begin{equation*}{\rm{LoD = LoB + 1}}{\rm{.645 \times S}}{{\rm{D}}_{{\rm{low\,concentration\,sample}}}}\end{equation*}$$

where SD represents standard deviation.

## RESULTS

### Development of a flow cytometry assay to detect HTLV-1 provirus in human lymphocytes

**Figure 2.**
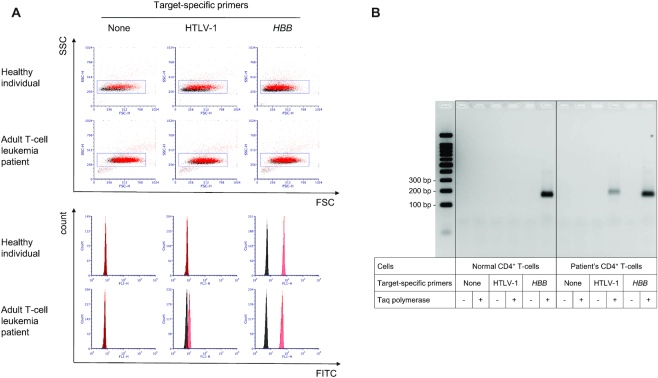
Human CD4^+^ T-cells containing HTLV-1 provirus can be detected by flow cytometry with high specificity. (**A**) Flow cytometry analysis of CD4^+^ T-cells in which *in situ* PCR was performed without target-specific primers and with primers targeting HTLV-1 or *HBB*. Histograms were created from populations gated in FSC/SSC dot plots. Results in the absence and presence of polymerase are coloured black and red, respectively. (**B**) Fluorescence imaging of an agarose gel. Cells described in A were digested using a protease and separated by agarose gel electrophoresis, and fluorescein in the gel was visualized.

We collected blood from ATL patients and a healthy individual, and isolated CD4^+^ T-cells (Figure 1B). We subsequently fixed and permeabilized cells, and mildly digested cellular proteins using a proteinase. Cells were then placed in a reaction mixture containing DNA polymerase, dNTPs, UniPrimer labelled with fluorescein, and target-specific forward and reverse primers. We added the tail sequence to both forward and reverse primers to increase the fluorescence signal. We included UniPrimers in all samples, but prepared samples without target-specific primers and with β-globin (*HBB*)-specific primers as negative and positive controls, respectively. After PCR amplification, reaction mixtures were diluted in phosphate-buffered saline (PBS) and cells were analysed by flow cytometry. We performed PCR in the presence and absence of Taq polymerase, and the results are coloured red and black, respectively (Figure 2A). If cells contain target sequences, they are amplified in the presence but not the absence of the polymerase, thereby resulting in a difference in fluorescence with and without polymerase. If cells do not contain target sequences, there is no amplification in the presence or absence of polymerase, resulting in no difference in fluorescence with and without polymerase.

We observed a single population in forward scatter (FSC) and side scatter (SSC) dot plots, and minimal difference in populations in the presence and absence of polymerase (Figure 2A). We assessed the fluorescence of most of the population using histograms, and observed no difference in fluorescence in the presence or absence of polymerase in samples without target-specific primers, indicating minimal noise generated from UniPrimers in the absence of target-specific primers. By contrast, we observed a marked difference in fluorescence in the presence and absence of polymerase in the presence of *HBB*-specific primers in cells from both the healthy individual and patient, indicating that *HBB* was present in both cells, and therefore amplified in the presence of polymerase. We also observed a clear difference in fluorescence in the presence and absence of polymerase with HTLV-1-specific primers in cells from the patient but not in cells from the healthy individual, indicating that HTLV-1 provirus was present in cells from the patient but not in those of the healthy individual.

To eliminate the possibility that fluorescence signals detected by flow cytometry were caused by non-specific PCR products, we digested cellular proteins after *in situ* PCR, performed electrophoresis, and detected fluorescein. We observed a single band of the expected size in all samples in which differences in fluorescence were observed by flow cytometry (Figure 2B), indicating that the fluorescence signals detected by flow cytometry were generated from specific but not non-specific PCR products. No band was observed in other samples in the presence of polymerase, indicating that background fluorescence (noise) was successfully prevented. We validated this method using samples from other ATL patients (Supplementary Figure S1). The results confirmed the successful detection of human lymphocytes containing HTLV-1 provirus by flow cytometry.

### Monitoring HTLV-1-infected tumour populations by flow cytometry

This assay could be used for monitoring ATL treatment. For example, we tested one ATL patient and found two populations in FSC and SSC dot plots (Supplementary Figure S2). Both populations consisted almost entirely of cells containing HTLV-1 provirus, indicating that two morphologically distinct tumour clones were present in the patient's blood. The patient received chemotherapy but did not respond, and therefore received peripheral blood stem cell transplantation. Flow cytometry analysis showed that neither clone was diminished following chemotherapy, but one clone was withdrawn after transplantation. The other population remaining after transplantation partially consisted of cells containing provirus, but was totally replaced with cells lacking provirus at 6 weeks after transplantation.

### Validation of the flow cytometry assay for the detection of single-copy DNA in human lymphocytes

**Figure 3.**
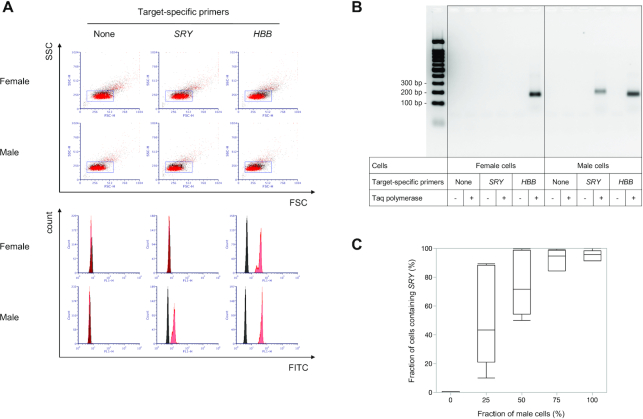
The fraction of human lymphocytes containing a single-copy gene can be estimated by flow cytometry. (**A**) Flow cytometry analysis of CD4^+^ T-cells in which *in situ* PCR was performed without target-specific primers and with primers targeting *SRY* (present as a single copy in male cells but not present in female cells) and *HBB*. Histograms were created from populations gated in FSC/SSC dot plots. Results in the absence and presence of polymerase are coloured black and red, respectively. (**B**) Fluorescence imaging of an agarose gel. Cells described in A were digested using a protease and separated by agarose gel electrophoresis, and fluorescein in the gel was visualized. (**C**) Box plot showing the percentage of positive cells in which *SRY* was detected by flow cytometry for five samples containing the indicated fraction of male cells. The line within the box represents the median, the ends of the box represent the first and third quartiles, and the whiskers extend to the 1.5× interquartile range of the box limits (*n* = 7 independent experiments).

We next targeted another single-copy DNA sequence in human lymphocytes. The *SRY* gene is located on the Y chromosome and is present as a single copy in male but not female cells (23). We collected blood from a healthy female and male, isolated CD4^+^ T-cells, and performed *in situ* PCR to amplify the *SRY* gene. The histograms generated in the presence of *SRY*-specific primers completely overlapped in the presence and absence of polymerase for female cells, but were distinctly separate in the presence and absence of polymerase for male cells (Figure 3A), demonstrating that the *SRY* gene is totally absent in female cells but present in male cells. We confirmed that fluorescence signals detected by flow cytometry were generated from specific PCR products using electrophoresis (Figure 3B). Thus, we successfully developed a flow cytometry assay capable of detecting single-copy genes in human lymphocytes.

### Optimization of experimental conditions

We found several factors that affected the specificity and efficiency of the *in situ* amplification reaction. Protease digestion was necessary to prevent non-specific *in situ* amplification. However, over-digestion caused cell lysis. Therefore, it is important to optimize protein digestion conditions such as protease concentration, incubation temperature, and time. The PCR cycle number and the concentration of cells in the PCR mixture affected the efficiency of *in situ* amplification. Fifty PCR cycles were required to detect every cell containing a single-copy target using our protocol (Supplementary Figure S3).

### Assessment of the flow cytometry assay for the quantification of lymphocytes containing single-copy target DNA

We next assessed the accuracy of the assay for quantifying cells containing a single-copy DNA target. We mixed female and male cells, and prepared five samples of each at male cell ratios of 0%, 25%, 50%, 75% and 100%. We then determined the fraction of cells containing the *SRY* gene by flow cytometry and compared the values to male cell ratios. Fractions of cells containing the *SRY* gene were roughly proportional to those of male cells (Supplementary Figure S4), but not perfectly proportional, and varied widely between experiments, particularly in samples containing low fractions of male cells (Figure 3C, Supplementary Table S2). The results indicate that flow cytometric quantification of cells containing a single-copy DNA target was not always accurate, especially when target-containing cells are present in low abundance.

We further estimated the limit of blank (LoB) and limit of detection (LoD) in the method. We mixed female and male cells, and prepared two samples of each at male cell ratios of 0% and 1%. We then measured the fraction of cells containing the *SRY* gene and determined the mean and standard deviation (SD) from nine replicates for each sample (Supplementary Figure S5 and Table S3). LoB and LoD were calculated as described in Materials and Methods. The blank and low concentration sample were defined as those containing 0% and 1% of male cells, respectively. LoB and LoD were estimated as 0.13% and 2.63%, respectively.

We further estimated the limit of blank (LoB) and limit of detection (LoD) of the method. We mixed female and male cells, and prepared two samples of each at male cell ratios of 0% and 1%. We then measured the fraction of cells containing the *SRY* gene and determined the mean and standard deviation (SD) from nine replicates for each sample (Supplementary Figure S5 and Table S3). The estimate of the LoB and LoD were calculated as described in Materials and Methods, where the blank and low concentration sample were defined as the sample containing 0% and 1% of male cells, respectively. The estimate of the LoB and LoD were 0.13% and 2.63%, respectively.

## DISCUSSION

*In situ* PCR is a technology with great potential, but its use is hampered by artefacts caused by non-specific amplification. Herein, we have overcome this issue by optimizing an *in situ* PCR protocol using UniPrimers. This approach eliminates fluorescent noise caused by non-specific *in situ* amplification, thereby raising the signal-to-noise ratio to a level sufficient for detecting single-copy DNA sequences by flow cytometry. In principle, this method allows the detection of any genomic sequences in freshly isolated human lymphocytes. The use of UniPrimers is of great benefit because any genomic sequence can be targeted solely by adding a tail sequence at the 5′ end of target-specific primers. This method may allow distinguishing single nucleotide differences in individual lymphocytes because UniPrimers can be combined with allele-specific PCR (24,25). Furthermore, multiple targets may be analysed by performing multiplex *in situ* PCR using UniPrimers with different combinations of fluorophore and tail sequences (24,25). Unlike FISH-based methods that are technically difficult and labour-intensive, the established flow cytometry method is simple. It could be potentially applied to detect cells that harbour genomic alterations such as aneuploidy, amplification, deletion, and gene fusions.

A few technical issues remain unsolved. First, quantification of cells containing the *SRY* gene was not sufficiently accurate, especially for rare populations. Wide variation in the percentage of *SRY*-positive cells was caused by fluorescence distributions of histograms. The histograms generated in the absence and presence of polymerase overlapped when the samples contained both female and male cells, and the fluorescence distributions were consistently narrow (Supplementary Figure S4); hence, even a slight shift could result in large differences in cell fraction. Accurate quantification could also be hampered by a transfer of PCR products from male cell into female cells. Further technical improvement is therefore needed for accurate cell measurement. Second, we optimized the protocol only for freshly isolated human lymphocytes. When using other cell types, experimental conditions should be optimized anew because optimal conditions differ widely between different cell types. The method may therefore not always be applicable to all cell types.

## Supplementary Material

gkaa515_Supplemental_File
