# Supplementary material for: Flow cytometry assay for the detection of single-copy DNA in human lymphocytes
Source: Nucleic Acids Res. 2020 Jun 16;48(15):e86. doi: 10.1093/nar/gkaa515 (PMC7470942; doi:10.1093/nar/gkaa515)
Supplement: gkaa515_Supplemental_File [file gkaa515_supplemental_file.pdf]

Supplementary Information

Flow cytometry assay for the detection of single-copy DNA in human lymphocytes

Naoki Uno, Norihito Kaku, Yoshitomo Morinaga, Hiroo Hasegawa and Katsunori Yanagihara

Department of Laboratory Medicine, Nagasaki University Graduate School of Biomedical Sciences. Nagasaki 852-8501, Japan

Supplementary Figures	2
Supplementary Figure S1	2
Supplementary Figure S2	3
Supplementary Figure S3	4
Supplementary Figure S4	5
Supplementary Figure S5	6
Supplementary Tables	7
Supplementary Table S1	7
Supplementary Table S2	7
Supplementary Table S3	7

Supplementary Figures

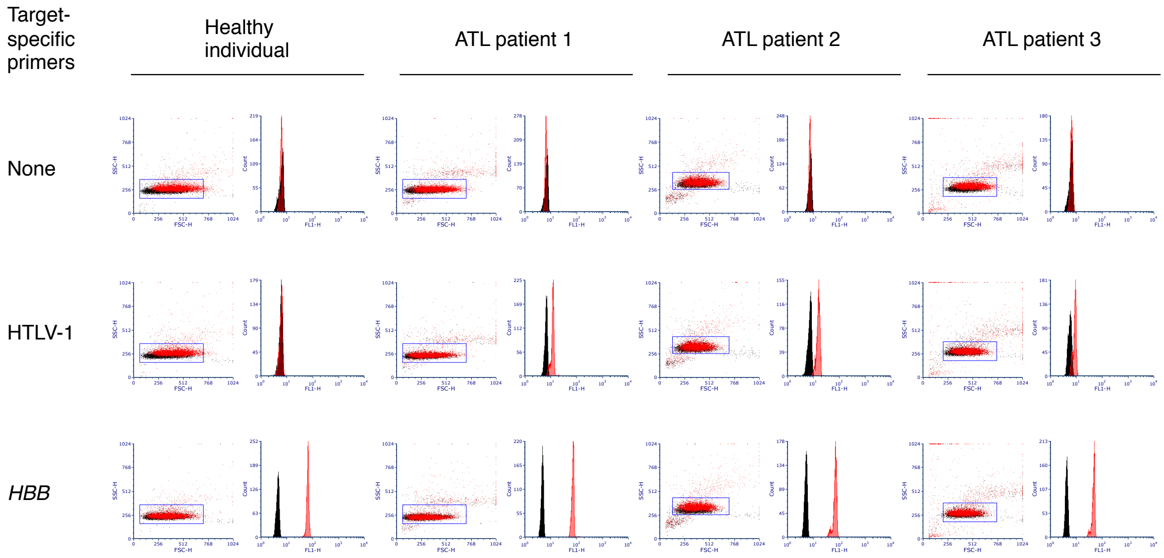

Supplementary Figure S1. Flow cytometry analysis of CD4^{+} T-cells derived from other ATL patients. *In situ* PCR was performed without target-specific primers and with the indicated target-specific primers in the absence and presence of polymerase in CD4^{+} T-cells separated from the blood of a healthy individual and three different ATL patients. Flow cytometry results in the absence and presence of polymerase are colored black and red, respectively.

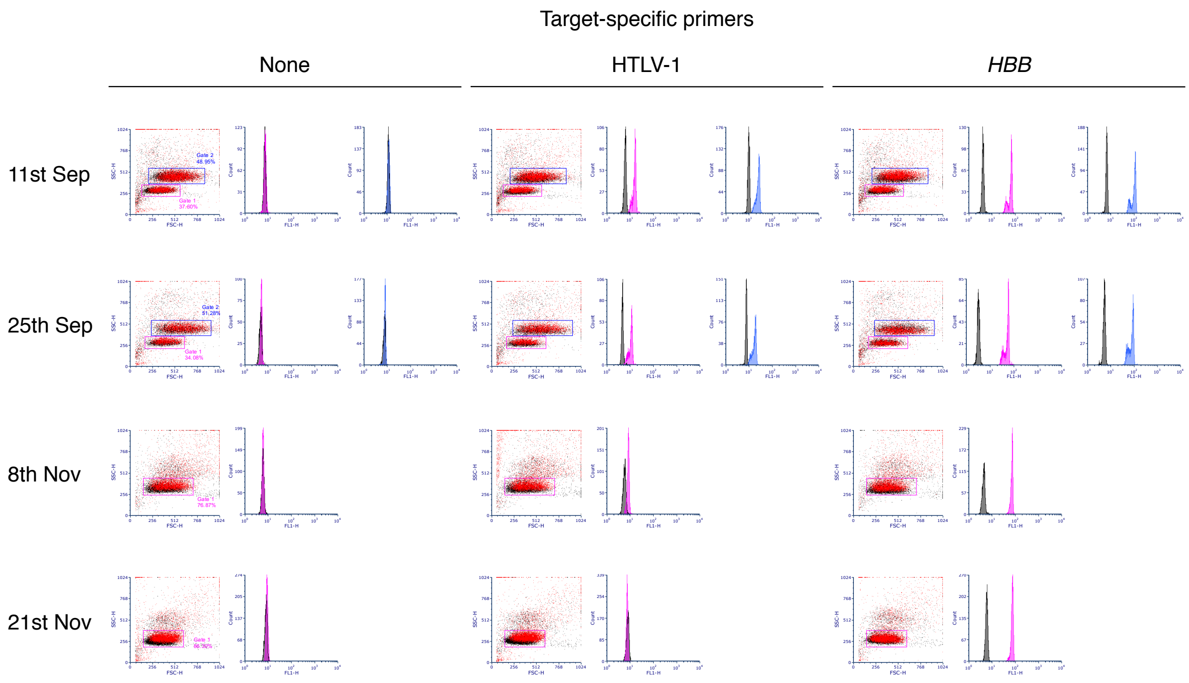

Supplementary Figure S2. Tracking of morphologically distinct HTLV-1$^+$ CD4$^+$ T-cell populations identified in blood from an ATL patient by flow cytometry. *In situ* PCR was performed without target-specific primers and with the indicated target-specific primers in the absence and presence of polymerase in CD4$^+$ T-cells separated from the blood of an ATL patient. Flow cytometry results in the absence and presence of polymerase are colored black and red, respectively, in FSC/SSC dot plots. Two distinct populations observed in FSC/SSC dot plots were gated and analyzed separately using histograms. Histograms obtained in the absence of polymerase are colored gray, and those in the presence of polymerase are colored pink and blue for the lower left and upper right populations, respectively. Flow cytometry analysis was performed at different time points to track each population. The patient received chemotherapy from the 4th to the 8th of September, followed by peripheral blood stem cell transplantation on the 13th of October.

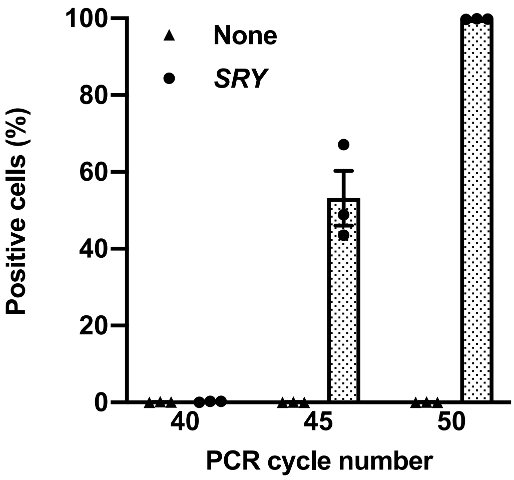

Supplementary Figure S3. Determination of the PCR cycle number required for detection of every cell containing a single copy target. *In situ* PCR was performed on $CD4^+$ T-cells derived from a healthy male in the absence of target-specific primers (filled triangle) and in the presence of *SRY*-specific primers (filled circle) for the indicated number of cycles. Following *in situ* PCR, cells were analyzed by flow cytometry. Error bars indicate standard error of the mean (n = 3).

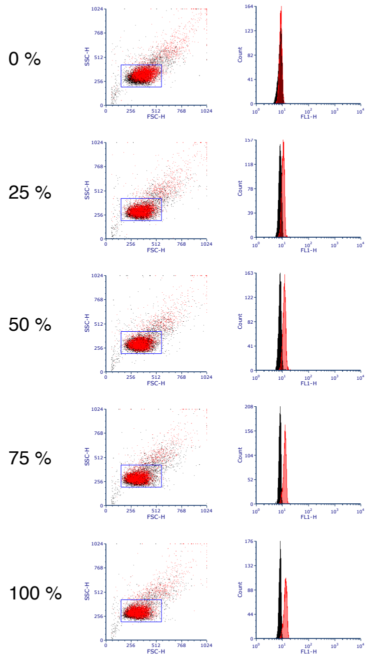

Supplementary Figure S4. Flow cytometry analysis of the fraction of cells containing the *SRY* gene. *In situ* PCR was performed to amplify the *SRY* gene in the absence and presence of polymerase for five samples containing male cells at the indicated ratios. A representative result of seven independent experiments is shown. Results in the absence and presence of polymerase are colored black and red, respectively.

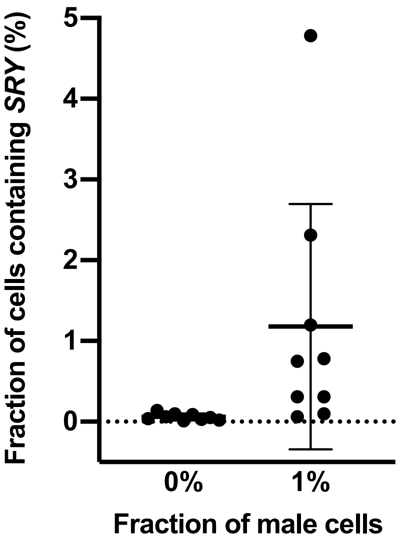

Supplementary Figure S5. Estimation of limit of blank and limit of detection. Percentage of cells containing the *SRY* gene determined by performing flow cytometry on samples containing 0% and 1% male cells. Error bars indicate standard deviation of the mean (n = 9).

Supplementary Tables

Supplementary Table S1. Oligonucleotide sequences of target-specific primers.

Primer name	Sequence (5′-3′)
HTLV-1 forward	ACTGAACCTGACCGTACAGGCAGATGACAATGACCATGAGC
HTLV-1 reverse	ACTGAACCTGACCGTACAGGGGAGACGTCAGAGCCTTATT
HBB forward	ACTGAACCTGACCGTACAGTGCATCTGACTCCTGAGGAGA
HBB reverse	ACTGAACCTGACCGTACACCTTGATACCAACCTGCCCAG
SRY forward	ACTGAACCTGACCGTACAGCTGGGATACCAGTGGAAAA
SRY reverse	ACTGAACCTGACCGTACATTCCGACGAGGTCGATACTT

All primers contain a tail sequence (underlined) that is identical to the 3′ end of the UniPrimer.

Supplementary Table S2. Five number summary of data in Figure 3C.

Fraction of male cells (%)	10	25	50	75	100
Maximum	9.970	89.29	99.57	99.47	99.87
3rd quantile	0.550	88.34	98.72	98.59	98.42
Median	0.070	43.40	71.64	94.57	95.80
1st quantile	0.060	20.97	54.29	84.45	91.17
Minimum	0.020	9.87	49.93	44.90	43.73

Supplementary Table S3. The mean and standard deviation of data in Supplementary Figure S5.

Fraction of male cells (%)	0	1
Mean	0.060	1.178
Standard deviation	0.042	1.521
